# Supplementary material for: Experimental evidence for circular inference in schizophrenia
Source: Nat Commun. 2017 Jan 31;8:14218. doi: 10.1038/ncomms14218 (PMC5290312; doi:10.1038/ncomms14218)
Supplement: Supplementary Information — Supplementary Figures, Supplementary Methods, Supplementary Discussion and Supplementary References [file ncomms14218-s1.pdf]

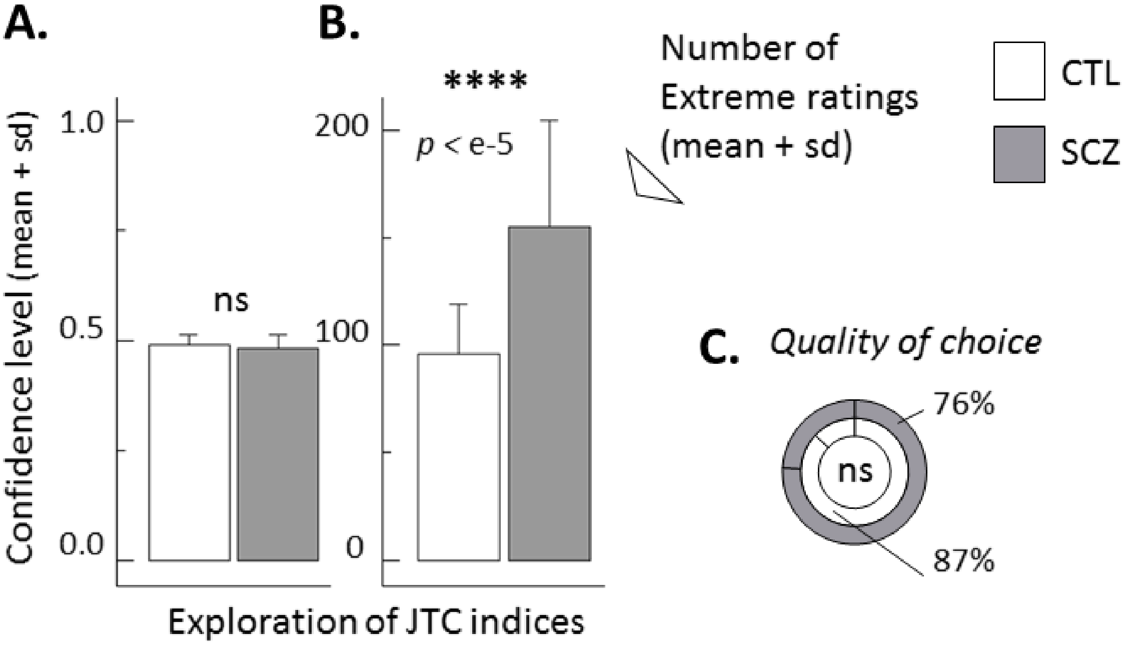

Supplementary Figure 1 | Evidence for a jumping-to-conclusions bias among patients with SCZ tested using the Fisher task.

Between-group differences were not observed in either the confidence levels associated with the lakes **(a)** or the *quality of choice* (**c**; i.e., whether participants' choices were in line with the optimal solution according to Bayes' theorem). In contrast, the patients with SCZ (n=25, grey) exhibited an increased number of extreme ratings relative to the matched healthy CTLs (n=25, white) **(b)**. The data are presented as the mean +/- SD.

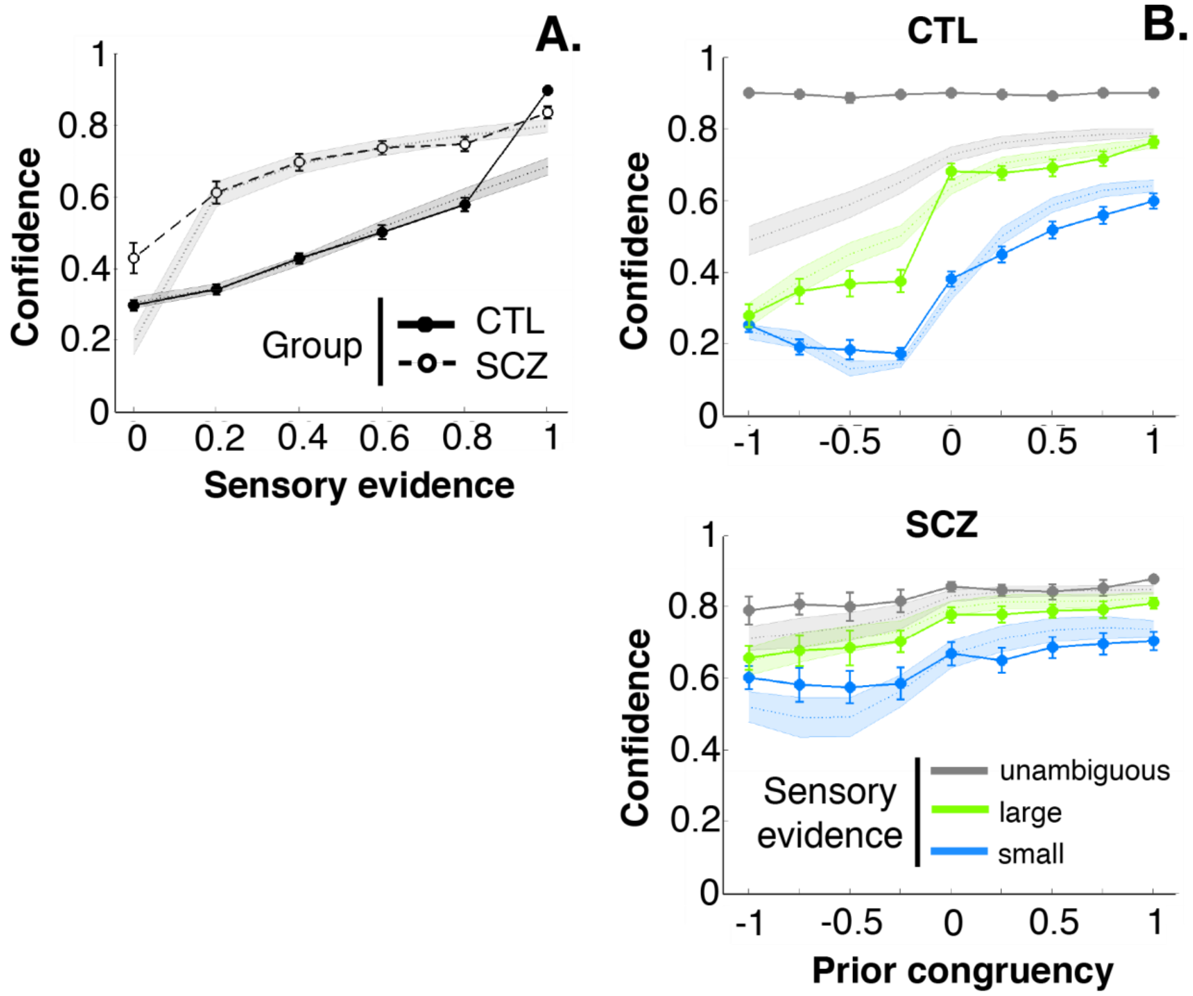

Supplementary Figure 2 | The predictions of the noiseless circular inference model in the CTL and SCZ groups' mean absolute confidence levels.

(a) Effects of sensory evidence. **(b)** Effects of prior congruency as a function or sensory evidence. Participants' mean performances+/-SDs are shown as dots with error bars. Model predictions+/-SDs are shown in the shaded areas. The predictions of the *circular inference model* fit CTL participants' performances across all trials, except those with unambiguous sensory evidence; in these cases, CTLs used a decision-making strategy based solely on sensory evidence. The predictions of the *circular inference model* fit the performance of participants with SCZ across all trial types, except those with zero sensory evidence, in which patients responded randomly at the extremes of the scale (but not in the middle). As Figure 1b shows, adding a small noise term (SD=0.03) to the likelihood accounted for this specific behaviour because, according to the model, the slope of the logit at approximately 0 is so large that any noise pushes the model predictions to the right or left of the scale.

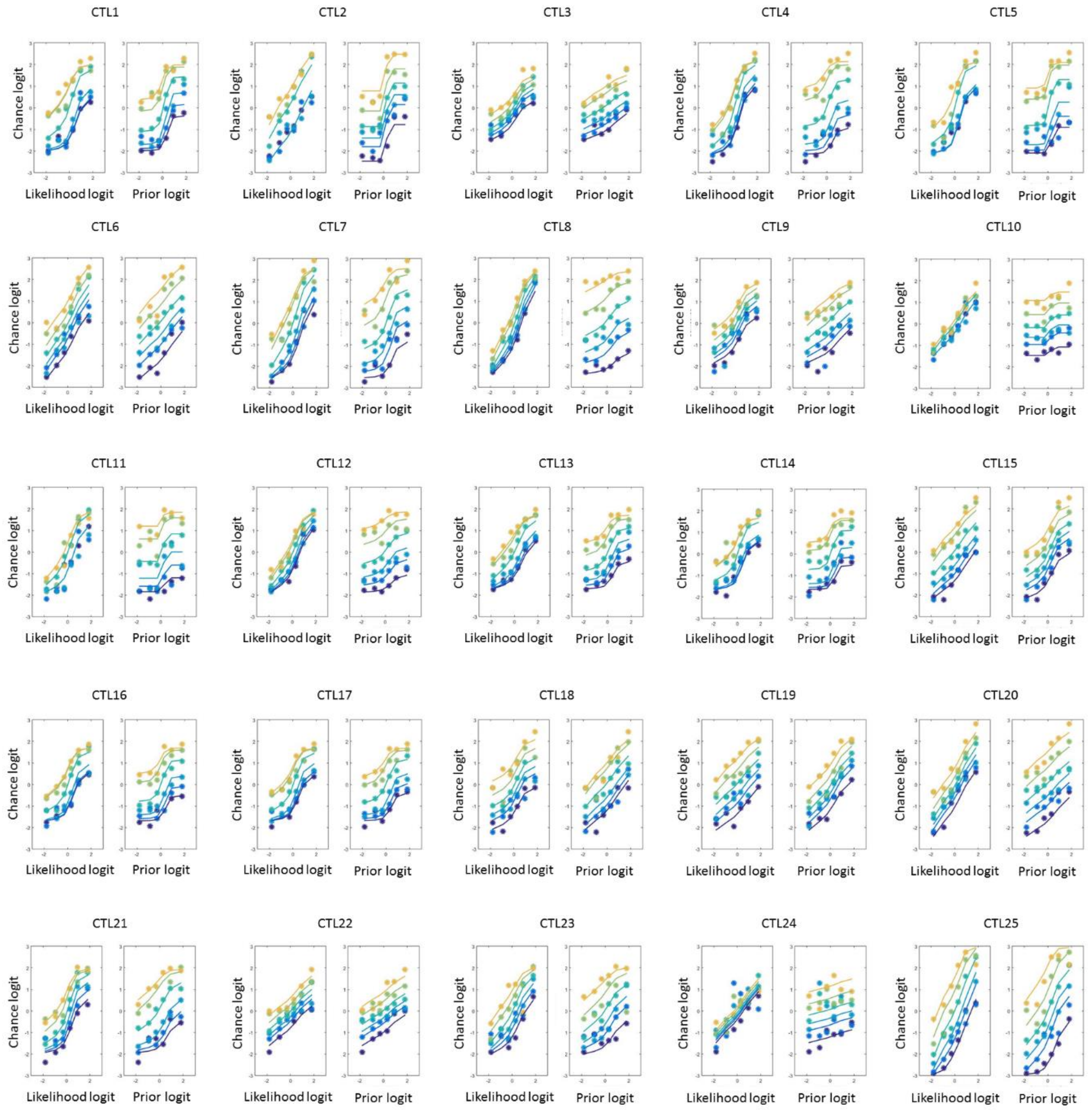

Supplementary Figure 3 | Average data at the subject level among CTLs (n=25).

The mean chance logit is plotted as a function of the likelihood logit or the prior logit for each participant in the CTL group. The likelihood and prior information are colour-coded (from dark blue to yellow) according to the true probability that the fish originated from one of the two lakes in the Fisher task. For each plot, the fit obtained using the *circular inference model* was overlaid as a solid line using the same colour code.

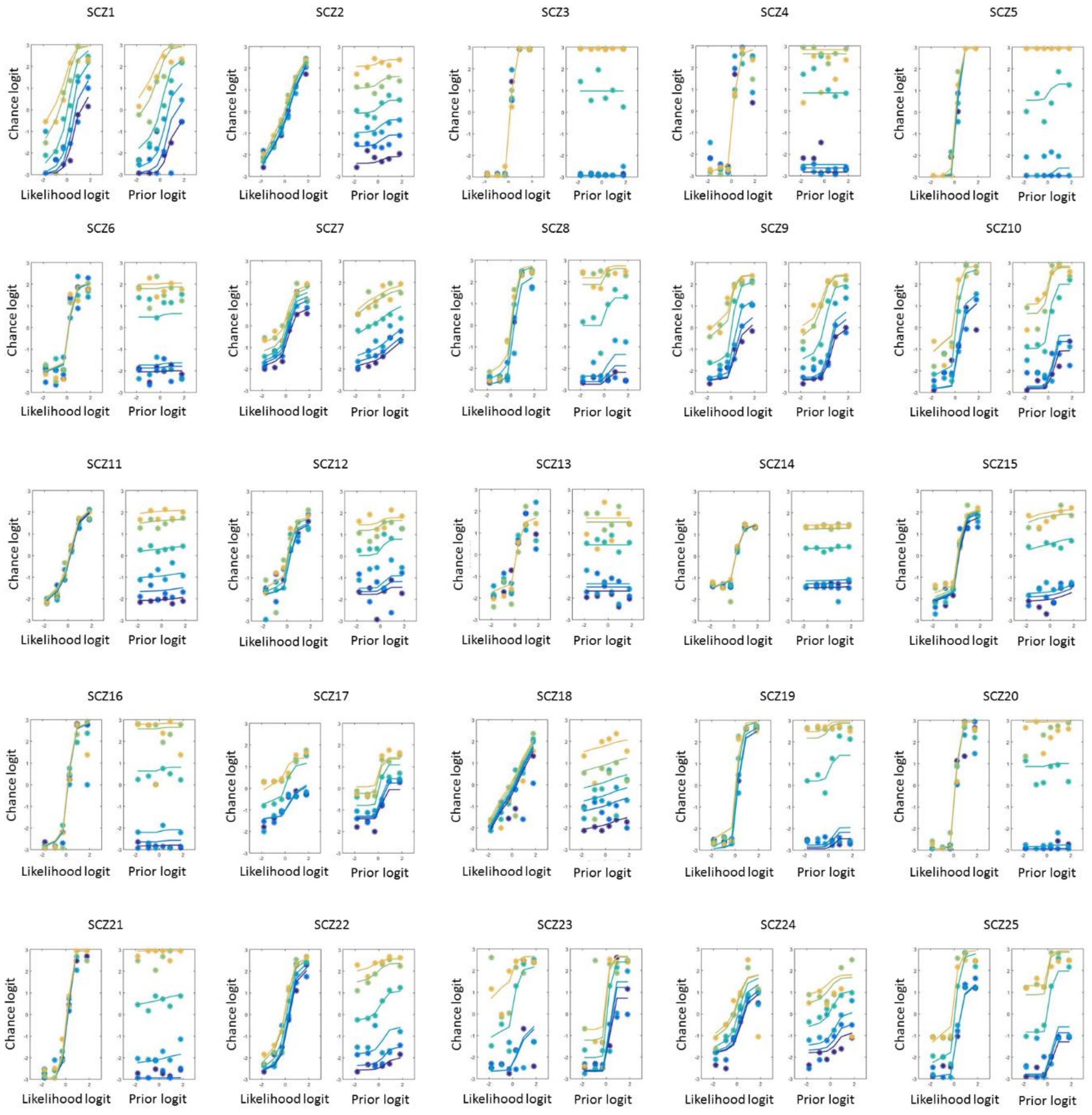

Supplementary Figure 4 | Average data at the subject level among patients with SCZ (n=25).

The mean chance logit is plotted as a function of the likelihood logit or the prior logit for each participant in the SCZ group. The likelihood and prior information are colour-coded (from dark blue to yellow) according to the true probability that the fish originated from one of the two lakes in the Fisher task. For each plot, the fit obtained using the *circular inference model* was overlaid as a solid line using the same colour code.

Supplementary Methods (MATLAB Codes)

fitall5_commented.m

```
% Loops over all subjects and fit parameters

Index=[w,x,y,z, etc.];
Nsubj=length(Index);

% For your information, these are the index of controls and patients. Datafiles (.mat files) are
% available on demands to the corresponding author.

% Control=[x, y, etc.]; %25 CTL
% Schz=[w, z, etc.]; %25 SCZ

param=zeros(71,5);

for k=Index(1:Nsubj)
   if k<10
     filename=sprintf('Subj00%d',k);
     else
    filename=sprintf('Subj0%d',k);
   end
   [epsp,epsl,wl,wp,feval]=fitdata5_commented(filename);
   param(k,:)=[epsl,epsp,wl,wp,feval];
end
```

fitdata5_commented.m

```
function [epsp,epsl,wl,wp,error]=fitdata5_commented(filename)

% This function extract the confidence of subject "filename" from his/her response on the scale, and
%fit the circular inference model.
% Subjects datafile will be provided on demands to the corresponding author.


% Load datafile
load([filename,'_OutputData.mat'])
Output=OutputMatrix;
sh=size(Output);

% Compute confidence from position on the scale
P=0.5;
angle=atan((Output(:,11)-1068*P)./-(Output(:,10)-792*P));
ang = mod(angle,pi)/pi;

% Initialize likelihood
likelihood=0.5*ones(sh(1),1);

% The problem with measuring angles this way is that PI + epsilon or -epsilon could be measured as
%2PI - epsilon. We avoid this by ensuring that we are on the correct side of the scale.
```

```
for it=1:sh(1)
   if (Output(it,10)-792*P)<0 && ang(it)<0.5
      ang(it)=1-ang(it);
   end

   if (Output(it,10)-792*P)>0 && ang(it)>0.5
      ang(it)=1-ang(it);
   end
      likelihood(it)=Output(it,4)/(Output(it,4)+Output(it,5));
end

prob=ang;
prior=Output(:,2)/100;
post=prior.*likelihood./(prior.*likelihood+(1-prior).*(1-likelihood));


% Select unambiguous trials
prior=prior(find(likelihood>0 & likelihood<1));
prob=prob(find(likelihood>0 & likelihood<1));
post=post(find(likelihood>0 & likelihood<1));
likelihood=likelihood(find(likelihood>0 & likelihood<1));

% Fit parameters from 100 different random initial conditions, and take the
% best fit (avoid ending up in a local minima).

for i=1:100
[param(:,i),fval(i)]=fminsearch(@(x) errpred5_commented(prob,prior,likelihood,x),[(rand-
0.5);(rand-0.5);(rand-0.5);(rand-0.5)],optimset('MaxFunEvals',10000));
end
[u,v]=min(fval);
param=param(:,v);
error=u;

% Extract optimal parameters
epsp=exp(param(1))./(1+exp(param(1)));
epsl=exp(param(2))./(1+exp(param(2)));
wl=exp(param(3))./(1+exp(param(3)));
wp=exp(param(4))./(1+exp(param(4)));
```

H_commented.m

```
%%%%%%%%%%%%%%%%%%%%%%%%%%%%%%%%%%%%%%%%%%%%%%%%%%%%%%%%%%%%
% THE H FUNCTION CHANGE THE GAIN (EPS X)BY ARTIFICIALLY MULTIPLYING MESSAGES PASSING
%%%%%%%%%%%%%%%%%%%%%%%%%%%%%%%%%%%%%%%%%%%%%%%%%%%%%%%%%%%%

function hout=H_commented(m,eps)

lpr=log((m(:,1)+0.000000001)./(1-m(:,1)+0.0000000001));

pcorr=(1+exp(-lpr*eps*60)).^(-1);
hout=[pcorr,1-pcorr];
```

errpred5_commented.m

```
function E=errpred5_commented(prob,p,l,param)

% Transform parameters from logit scale to probability scale:

   %1. strength of descending loops
epsp=exp(param(1))./(1+exp(param(1)));

   %2. strength of ascending loops
epsl=exp(param(2))./(1+exp(param(2)));

   %3. sensory (likelihood) weight
wl=exp(param(3))./(1+exp(param(3)));

   %4. prior weight
wp=exp(param(4))./(1+exp(param(4)));

% Keeps reported confidence between about -4 and 4 (otherwise perceived logits would be
%undefined if subjects clicked even slightly outside the scale, plus logits are very unreliable when
%prob is close to 0 or 1(they become extremely sensitive to any small fluctuations, e.g. "motor
%noise").
% prob=min(max(prob,0.018),0.982);
prob=min(max(prob,0.01),0.99);


% "pred5" predicts the perceived logit using circular inference model
pr=pred5_commented(p,l,epsp,epsl,wl,wp);

% Bounds predicted confidence
% pr=min(max(pr,0.018),0.982);
pr=min(max(pr,0.01),0.99);

% compute logits from probabilities
lprob=log(prob./(1-prob));
lpred=log(pr./(1-pr));

% Compute fitting errors. Note that there is a cost on large amounts of loops
%(0.01*epsp.^2+0.01*epsl.^2).
% This prevents "degenerate" solutions with prior weighting around 0 and epsp = very large. Indeed,
%if prior weight is 0,
% then descending loops could be arbitrarily (and artificially) large, without changing any model
%predictions. Quadratic cost insures that epsp = 0 in those cases. Reported statistical results are
%largely insensitive to the weighting (here, 0.01) of this cost.

E=sum((lpred-lprob).^2)+0.01*epsp.^2+0.01*epsl.^2;

% Note: we chose to use mean squared error on logits because we observed that the bounded
%perceived logit are close to Gaussian distributed with condition-independent variance.
% KL divergence is a better measure of probability distance, unfortunately it is extremely sensitive to
%any small fluctuations around 0 or 1, and thus, to motor noise. On the other hand, the distribution
%of confidence (prob) is extremely non Gaussian, rendering MSE fitting inappropriate. The model
%fitted with MSE on logits has the highest BIC score.
```

pred5_commented.m

```
function P=pred5_commented(p,l,epsp,epsl,wl,wp)

% To avoid potential numerical issues (e.g. infinite likelihood logits or confidence logits) this
%prediction is computed in terms of the probability and not logits.
% Turned into logits, it is equivalent to the circular inference model equations reported in the text.

% Turn likelihood logits into probabilities of "right" and "left" lake.
m0=[p,1-p];

% Turn prior logits into probability
ml=[l,1-l];

% Turn prior and likelihood weights into conditional probability matrix.
Gp=[1-(1-wp)*0.5 (1-wp)*0.5
   (1-wp)*0.5 1-(1-wp)*0.5];
Gl=[1-(1-wl)*0.5 (1-wl)*0.5
   (1-wl)*0.5 1-(1-wl)*0.5];

% Apply circular inference model. "H" is a function that turns "alpha * logits" into a probability
%format (see "H_commented.m").

bc=(((ml).*(H_commented(m0,epsp)*Gp).*(H_commented(ml,epsl)*Gl))*Gl).*(((m0).*(H_commented
(ml,epsl)*Gl).*(H_commented(m0,epsp))*Gp)*Gp);
P=bc(:,1)./(bc(:,1)+bc(:,2));
```

Supplementary Discussion

The particular case of unambiguous trials

Thus far, we considered the model prediction in ambiguous trials (i.e., with finite likelihood logits). In unambiguous trials, the lakes contain only black or red fishes, making it normally impossible for the fish to come from one of the two lakes. In those trials, the participants should use a very simple “rule based” strategy, and click systematically at the corresponding extremity of the scale, as they were encouraged to so during the instructions (see translation in supplementary information). Interestingly, we found that CTLs and patients did not treat these trials in the same fashion. CTLs clearly used a decision-making strategy solely based on sensory evidence, regardless of how they trust sensory evidence and prior in ambiguous trial (i.e., regardless of w_p and w_s). This observation implies

that the probabilistic model cannot capture their response in unambiguous trials (**Supplementary Fig. 2b, top panel**). In contrast, the patient group confidence was lower in unambiguous trials and still affected by the prior (**Supplementary Fig. 2b, bottom panel**). The probabilistic model captures this behaviour (**Fig. 1c**), suggesting that patients use the same probabilistic strategy in ambiguous and unambiguous trials. It may be that patients have more difficulties than CTLs in switching between two different strategy, or that they interpret the instruction differently.

The particular case of a no-reverberation model

We also tested a "no-reverberation" model, Lc = F(Ls + F(AsLs,Ws)) + F(Lp + F(ApLp,Wp)). Despite having the same number of free parameters, its performance in terms of BIC score is markedly weaker than the model with reverberation, i.e., "circular inference" (BIC_reverberation – BIC_no reverberation = -95). This "no-reverberation" model predicts that the subject's confidence would be the sum of two independent functions, one depending purely on the likelihood, the other depending only on the prior. In particular, it predicts that the slopes of all the sigmoids in **Figure 3** would be identical. This is clearly not the case, i.e., the confidence is influenced more strongly by the likelihood when the prior is non-informative (Lp = 0) that when the prior is strongly positive or negative. Vice-versa, the prior influences more the confidence when Ls is small. Note that while the effect appears moderate in **Figure 3a,b** as a result of averaging heterogeneous subject data, it is much more evident in individual subjects (see **Figure 3c,d,e,f**, and **supplementary Figures**).

Jumping to conclusions and alternative interpretations

In the *jumping-to-conclusions* literature, delusions are often described as false beliefs that are not changed after exposure to contrary evidence, a phenomenon known as a *bias against disconfirmatory evidence*[1]. In the absence of quantitative model fits, we were unable to determine whether this bias arose because the weight of later samples was weaker or because the participants had already derived high levels of confidence from the previous samples and therefore required more evidence to update their level of confidence. This result demonstrates the importance of using

parameterised probabilistic inference models to compare CTLs with patients with SCZ as well as the importance of comparing both groups with ideal Bayesian observers.

Other studies have used a similar strategy and fit a parametric Bayesian decision model to participant choices using a variant of the beads task paradigm[2 3]. However, these authors assumed that the patients with SCZ would be unimpaired with regard to Bayesian integration per se; this assumption constitutes a major difference from the present framework. Their results suggested that decision noise was greater in patients with SCZ than in CTLs[2]. This result might have led the patients with SCZ to lower their decision threshold[3], an optimal strategy in such a context, resulting in jumping to conclusions. In simpler terms, it is not worth waiting to accumulate more data (or more samples) if the decision process itself is unreliable.

These interpretations are not completely consistent with our data. First, the patients with SCZ in the current study were not required to make decisions; rather, they merely reported their confidence level. This confidence should have been lower when the participants assumed that the sensory information was less reliably integrated. Second, we did not observe any evidence for higher decision noise in patients with SCZ. In fact, the patients with the highest level of ascending loops were able to perfectly discriminate slight differences in the proportions of the 50 fishes present in each of the two lakes (0.4 versus 0.6) and made no errors (defining “error” as choosing the “wrong” side of the scale). In this sense, these patients were more reliable than CTLs, whose reported confidence varied in such trials.

Another hypothesis that has been proposed to account for jumping to conclusions is a bias towards a hypersalience of evidence (i.e., in favour of the actual choice)4. This hypothesis is compatible with the “ascending loops” hypothesis of the circular inference framework: Ascending loops combine sensory evidence with their own top-down predictions, even in the absence of an independent source of prior information. In turn, the participant’s choice can be correlated with (or even the cause of) this top-down prediction.

Supplementary References

1. Woodward TS, Moritz S, Cuttler C, Whitman JC. The contribution of a cognitive bias against disconfirmatory evidence (BADE) to delusions in schizophrenia. *Journal of clinical and experimental neuropsychology* **28**, 605-617 (2006).
2. Moutoussis M, Bentall RP, El-Deredy W, Dayan P. Bayesian modelling of Jumping-to-Conclusions bias in delusional patients. *Cogn Neuropsychiatry* **16**, 422-447 (2011).
3. Averbeck BB, Evans S, Chouhan V, Bristow E, Shergill SS. Probabilistic learning and inference in schizophrenia. *Schizophr Res* **127**, 115-122 (2011).
4. Speechley WJ, Whitman JC, Woodward TS. The contribution of hypersalience to the "jumping to conclusions" bias associated with delusions in schizophrenia. *J Psychiatry Neurosci* **35**, 7-17 (2010).
